# Physical and Clinical Evaluation of Hip Spica Cast applied with Three-slab Technique using Fibreglass Material

**DOI:** 10.5704/MOJ.1611.008

**Published:** 2016-11

**Authors:** KM Bitar, ME Ferdhany, EI Ashraf, A Saw

**Affiliations:** Department of Orthopaedic Surgery, University Malaya Medical Centre, Petaling Jaya, Malaysia

**Keywords:** DDH, hip spica cast, paediatric femur fracture, closed reduction

## Abstract

**Introduction:** Hip spica casting is an important component of treatment for developmental dysplasia of the hip (DDH) and popular treatment method for femur fractures in children. Breakage at the hip region is a relatively common problem of this cast. We have developed a three-slab technique of hip spica application using fibreglass as the cast material. The purpose of this review was to evaluate the physical durability of the spica cast and skin complications with its use.

**Methodology:** A retrospective review of children with various conditions requiring hip spica immobilisation which was applied using our method. Study duration was from 1st of January 2014 until 31st December 2015. Our main outcomes were cast breakage and skin complications. For children with hip instability, the first cast would be changed after one month, and the second cast about two months later.

**Results:** Twenty-one children were included, with an average age of 2.2 years. The most common indication for spica immobilisation was developmental dysplasia of the hip. One child had skin irritation after spica application. No spica breakage was noted.

**Conclusion:** This study showed that the three-slab method of hip spica cast application using fibreglass material was durable and safe with low risk of skin complications.

## Introduction

Hip spica casting is a common treatment method for a variety of conditions that requires immobilization of the femur and pelvis^1^. They include femur fracture in young children, proximal femur osteotomy, septic arthritis of the hip and developmental dysplasia of the hip (DDH). Successful treatment of these conditions is dependent on the physical integrity of the spica cast, and morbidity related to its prolonged application. Most publications reporting the use of hip spica cited the technique described by Kumar in 1981 where multiple strips of plaster of paris (POP) slabs were applied across the joints and limbs^2^.

Breakage of the cast, especially at the femoral pelvic junction, is a common problem with the use of hip spica cast with POP material; this why some authors developed modifications of the standard technique to improve the durability, including application of a cross bar connecting both the lower limb components^3^. We modified the Kumar’s technique by using three-slabs across the hip joints and relied on them to provide the stability for the whole spica cast. By modifying the placement of these slabs, we hoped to improve the strength of spica cast across the femoral pelvic junction to protect against breakage.

Both plaster of paris (POP) and synthetic fiberglass material have been used for hip spica casting^4^. For the last three years, we converted to using fibreglass material to further improve the physical durability of the cast and reduce the weight of the whole construct. POP has good moulding capability, but has been shown to be mechanically inferior compared to fibreglass^5^. Furthermore, attempt to increase its’ strength by using more cast material would result in a heavier cast which could be inconvenient to the child and the parents.

The purpose of this study was to evaluate the physical outcome of hip spica applied with our modified technique using fibreglass as cast material, and evaluate the clinical outcome of this procedure including clinical complications and short term stability of the hip after removal of cast.

## Materials and Methods

We performed a retrospective study on consecutive cases of hip spica castings from 1st January 2014 until 31st December 2015. All children regardless of age and indication for hip spica casting were included in the study. For children with hip instability, cast change would be performed at about four weeks after application to evaluate the hip stability both clinically and with C-arm imaging. The second hip spica would be removed about eight weeks later in the paediatric out-patient clinic with no anaesthesia. We routinely performed plain radiography of the hips about two weeks after cast removal, and review about 6 months for the next one of two years. We traced medical records for clinical review, and evaluated plain radiographs taken before and during hip spica cast applications, and at least 2 weeks after cast removal. Being a retrospective study, we collect additional feedbacks via phone interviews with the parents on problems they experience during spica cast application and overall satisfactions of the procedure.

**Fig. 1:**
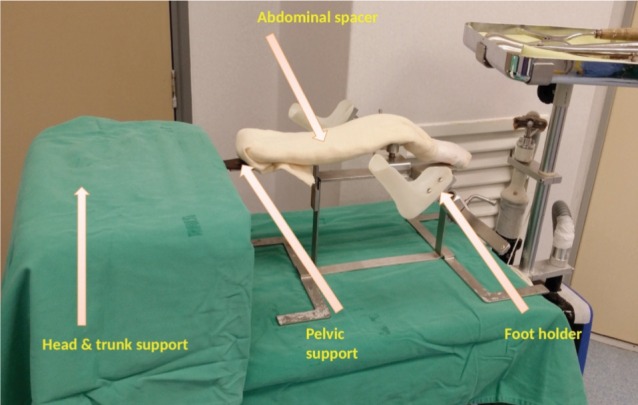
Holder for the trunk and lower limbs for hip spica application in young children. Container box for the device was used as the platform to support the head and thoracic spine.

We used a locally fabricated holder to position the trunk and limbs of the child before spica cast application (Fig. 1). The foot holder would allow both the hips to be positioned at 90 degrees flexion, and about 60 degrees on abduction, depending of range of the safe zones. The holder did not allow us to position the limbs in internal rotation. In general, hip spica cast application involved the use of 6 rolls of fibreglass cast material. All the spica cast applications were performed under general anaesthesia. As the first step, a longitudinal padding was placed along the chest and abdomen. The trunk and limbs were covered with 2 layers of webril with or without additional layer of stockinette (Fig. 2a). The first layer of cast material was applied over the trunk. Next, two slabs were applied across the anterior and posterior aspects of both hips in the shaped of the alphabet “U”. Front ends of both the slabs would cross the midline anteriorly at the level of umbilicus, and the back ends of these slabs would lie longitudinally along the posterior aspect of the trunk cast (Fig. 2b). A third slab would be placed across the posterior aspect of the trunk crossing the back ends of the U shaped slabs at right angle posteriorly, and swing across lateral aspect of both hips to the anterior aspects of the groins. (Fig. 2c). After this step, additional casts will be applied over both the lower limbs either to just above the knee for the unaffected side, or just above the ankle for the affected limb. Additional cast may be used to strengthen other parts of the cast if necessary. In average, for children below the age of 3 years old, we used about 6 to 7 rolls of fibreglass cast material (3-inches or 5-inches width).

**Fig. 2a:**
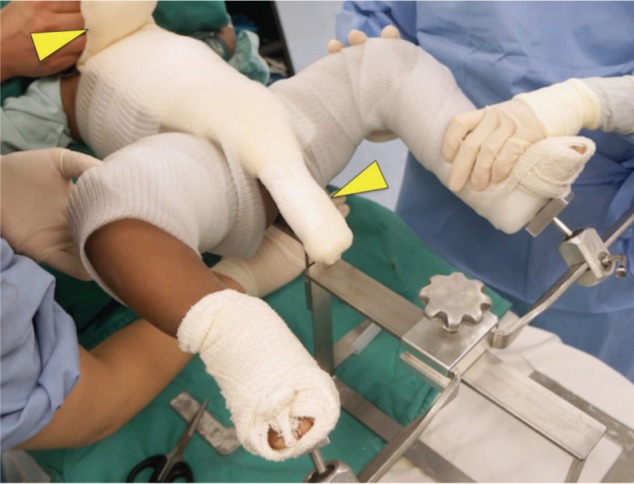
Two layers of inner liner applied before fibreglass cast. Yellow arrow indicate a spacer made from filling a narrow stockinette with multiple layers of cotton bandages. The spacer is used to ensure adequate room with the cast for abdominal expansion, and maintain adequate opening for perineum hygiene.

**Fig. 2b:**
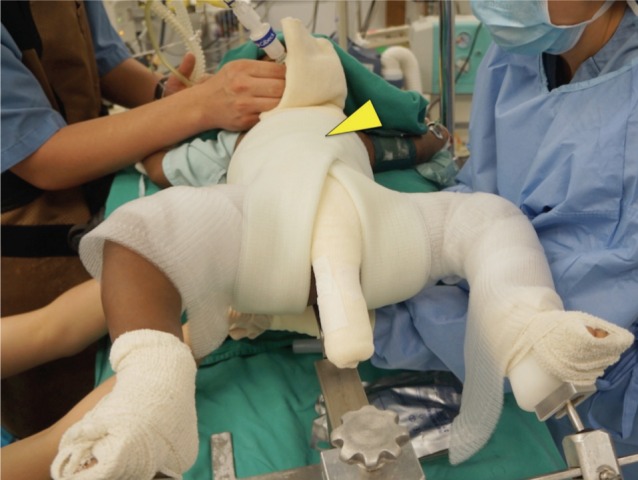
Anterior ends of the two slabs (1st and 2nd slabs) crossed the midline at the level indicated by the yellow arrow. Posterior ends were applied along the long axis of the trunk.

**Fig. 2c:**
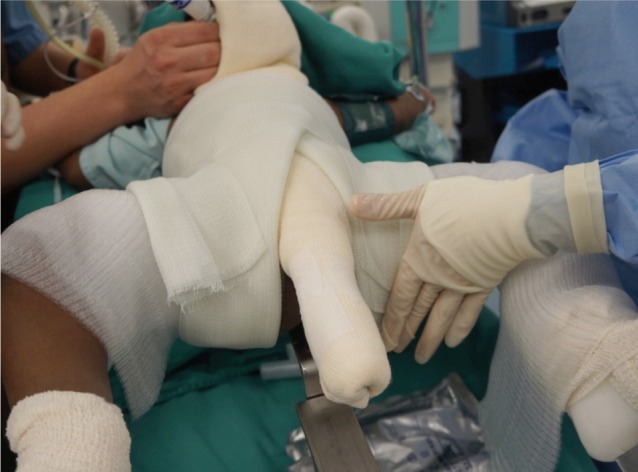
The long posterior slab (3rd slab) was applied superficial to the posterior ends of the first two slabs and wind around the body to end at the anterior aspect of the hips.

Children who requested for further follow up and cast removal in another institution were excluded from this study. Main outcome parameters included any form of skin complication, improper fitting that requiring cast modification or trimming and breakage of the cast. Failure of treatment was defined as subluxation / dislocation of hip based on clinical and radiological evaluations at least two weeks after cast removal, and malunion.

## Result

Twenty-one children underwent hips spica application during the study period. The mean age was 2.2 years (5 months to 4 years). There were 13 girls and 8 boys, with a female to male ratio of 1.6: 1. (Table I). Forty-one hip spica were applied for the 21 children. The most common indication for hip spica application was developmental dysplasia of the hip (DDH) (n=15), septic hip dislocation (n=3), syndromic hip dislocation (n=2) and pathological femur fracture (n=1). We included a child (case 17) with osteogenesis imperfecta who fractured her femur and was treated with hip spica cast application using the same technique, except that we need additional moulding for the affected thigh while the cast was setting. She has only one cast application for 2 months.

**Table I:** Number of casts according to underlying conditions

Underlying condition	No. of children	No. of casts
DDH	15	30
Septic arthritis	3	6
Syndromic hip dislocation	2	4
Femur fracture	1	1
Total	21	41

There was no hip spica breakage noted in this series. One child had a pressure sore at her left groin due to skin irritation by the edge of the cast at the perineal opening. This was noticed 3 days after spica application. The cast was re-applied and subsequently the sore resolved uneventfully. For the 20 children with hip instability, post-operative radiograph and clinical examination did not show any evidence of instability. Subsequent progress of the conditions was beyond the scope of this study.

## Discussions

With our method of hip spica application using fibreglass material, we did not record any breakage of the spica during the period of application. Mechanical failure of hip spica, especially breakage at the thigh-trunk junction is one of the most common failures of this treatment technique. In a study comparing hip spica casts with and without additional bar across the limbs, Hosalkar *et al*^3^ reported 11% of premature hip spica breakage and all of them were in the group without cross bar. Although they reported that the cross bars did not hamper toileting and handling, time for cast application and removal might be longer, and more cast material might be necessary. We decided to use fibreglass cast material due to its faster setting time, superior mechanical strength^4,5^ and ability to retain 70% to 90% of initial strength upon contact with water^6^. Hybrid POP-fibreglass casts^7^ have been recommended to improve the durability and reduce the cost. However, they were still heavier and not as strong as the fibreglass only cast. In addition, radiolucency of fibreglass material allows more accurate assessment of hip stability after cast application compared to POP only or hybrid casts. Our study showed that combination of the three-slab technique and use of fiberglass material could provide us with hip spica casts that were light, radiolucent and strong enough to withstand physiological loads for at least two months.

Skin irritation in the form of abrasion, pressure sore, and infection / infestation are common problems related to plaster cast application, especially for prolonged use. In a study on 297 patients with 300 hip spica cast for femur fractures, DiFazio *et al*^8^ reported that 77 (28%) patients had skin complication. Among these patients, some required unscheduled cast change under anestehsia (31%), early cast bivalving (44%), or cast trimming (25%). We have one child (case 21) with abrasion over the inner thigh corresponding to the un-intentional edge inversion of the perineum opening. Since trimming of the edge might end up with sharp edge of fiberglass material, we decided to reapply the hip spica under anaesthesia. Subsequent recovery has been uneventful. Our results showed a relatively low rate of skin complications within our method of hip spica cast application.

The main limitation of our study would be the small sample size. Being a retrospective study, possibility of reporting bias for skin complications may be possible. However, it would be not very likely for the primary outcome of cast breakage to be missed. Although all the unstable hips were reduced after cast removal based on clinical and radiological evaluations, subsequent subluxation or dislocation may still be possible with longer follow up. However, long-term outcome of unstable hip is influenced by many other factors including type of underlying pathology, age at presentation, and type of surgical intervention. This is not the primary outcome we are investigating in this study.

## Conclusion

This study showed that the three-slab method of hip spica application using fibreglass material was reliable to provide immobilization for the femur and hip joint with low risk of cast breakage or skin complications. Low weight and radiolucency were additional advantages for this technique.

## References

[1] Terjesen T, Halvorsen V (2007). Long-term results after closed reduction of late detected hip dislocation: 60 patients followed up to skeletal maturity. Acta Orthop..

[2] Kumar S (1981). Hip spica application for the treatment of congenital dislocation of the hip. J Pediatr Orthop..

[3] Hosalkar HS, Jones S, Chowdhury M, Chatoo M, Hill RA (2003). Connecting bar for hip spica reinforcement: does it help?. J Pediatr Orthop B..

[4] Adkins L (1997). Cast changes: synthetic versus plaster. Pediatr Nurs..

[5] Wytch R, Mitchell C, Wardlaw D, Ledingham W, Ritchie I (1987). Mechanical assessment of polyurethane impregnated fibreglass bandages for splinting. Prosthet Orthot Int..

[6] Berman A, Parks B (1990). A comparison of the mechanical properties of fiberglass cast materials and their clinical relevance. J Orthop Trauma..

[7] Charles MN, Yen D (2000). Properties of a hybrid plaster-fibreglass cast. Can J Surg..

[8] DiFazio R, Vessey J, Zurakowski D, Hresko MT, Matheney T (2011). Incidence of skin complications and associated charges in children treated with hip spica casts for femur fractures. Pediatr Orthop..

